# Research on Material Optimization of CSM Method Structures in Highly Weathered Strata

**DOI:** 10.3390/ma19071287

**Published:** 2026-03-24

**Authors:** Yifan Xie, Haitao Liu, Hao Wen, Chuangui Sun, Yong Chang, Qiang Feng, Lianzhen Zhang, Hongbo Wang

**Affiliations:** 1 College of Civil Engineering and Architecture, Shandong University of Science and Technology, Qingdao 266590, China; 15865470329@163.com (Y.X.); wh990323@163.com (H.W.); 15020344356@163.com (C.S.); fengqiang@sdust.edu.cn (Q.F.); 2 China Railway First Group No.5 Engineering Co., Ltd., Baoji 721006, China; 15269391279@163.com (H.L.); changyong202602@163.com (Y.C.); 3 College of Pipeline and Civil Engineering, China University of Petroleum, Qingdao 266580, China; zhanglianzhen@upc.edu.cn

**Keywords:** CSM method, highly weathered strata, foundation support, basalt fiber–reinforced soil–cement material, H–section steel

## Abstract

To address the challenges of low strength and poor impermeability of soil–cement walls formed with ordinary cement materials when applying the CSM (Cutter Soil Mixing) method in highly weathered strata, this study carried out structural optimization by combining the CSM method with H–section steel. This optimization effectively resolves issues such as low efficiency and high cost associated with the CSM method integrated with cement–filled piles. Meanwhile, using ordinary Portland cement as the base material, basalt fiber, sodium bentonite, and fly ash were added to investigate the influence of each component on the performance of the new composite. A novel CSM material suitable for highly weathered strata was developed, which exhibits excellent mechanical strength and impermeability. The optimal mix proportion of the soil–cement material was determined as follows: basalt fiber 0.5%, fly ash 15%, and sodium bentonite 3%. This research provides a quantitative basis for the efficient and economical application of the CSM method in highly weathered strata.

## 1. Introduction

With the acceleration of urbanization in China, the scale of underground space development and utilization continues to expand, placing higher demands on foundation pit support technologies. Among various support techniques, the CSM (Cutter Soil Mixing) method has been widely applied in engineering fields such as cutoff walls, support structures, and ground improvement, due to its ability to form continuous walls by uniformly mixing in situ soil with cement slurry through a simultaneous cutting and mixing process. However, limited by the insufficient curing strength, poor impermeability, and high cost of ordinary cemented soil walls, the economic efficiency and construction speed of projects have been severely impacted. The current composite process that combines the traditional CSM method with cast–in–place piles struggles to meet the dual demands of modern engineering for both construction efficiency and economic viability, nor can it effectively address the core issues of inadequate mechanical properties and impermeability of cemented soil walls in foundation pit support. Therefore, there is an urgent need to develop structurally optimized solutions and to research new cementitious materials.

Traditional support techniques, including deep mixing piles and TRD (Trench Cutting Mixed Deep Wall) methods, are frequently associated with limitations such as inadequate strength, inconsistent waterproofing performance, and poor adaptability to heterogeneous rock formations [1,2,3,4]. In highly weathered strata, conventional cemented soil mixtures are unable to fulfill both mechanical strength and impermeability requirements concurrently, resulting in elevated construction costs and potential safety risks [5]. Currently, scholars have achieved certain results in research on its design theory, construction technology, structural optimization, effect inspection, and theoretical analysis. Brunner W G introduced the innovative CSM technology for constructing retaining walls and cutoff walls, comparing its advantages over traditional soil mixing methods [6]. Successfully implemented CSM theory in practical engineering applications, with full consideration of the actual project environment [7,8,9]. In engineering practice, the CSM method was further optimized by introducing section steel [10,11,12]. It is proposed to replace traditional cast–in–place concrete piles with H–section steel inserted into the TRD cemented soil to form an integrated support structure. This approach not only addresses the issue of insufficient strength of TRD but also enables the recycling of H–section steel, thereby reducing engineering costs [13]. Nevertheless, existing approaches have largely concentrated on structural improvement, with insufficient attention paid to cost–effectiveness, making them unsuitable for cost–sensitive large–scale underground projects.

Building on studies of the solidification mechanism of cement–treated soil and fiber–reinforced cement–treated soil, this research investigates the impact of fiber materials on the physical and mechanical properties of cement–based composites through the incorporation of fibers into the cementitious material [14,15,16,17]. Through the investigation of cemented soil, it was found that the solidification mechanism of the improved cemented soil includes the hydration reaction of cement, the bridging and crack resistance effects of fibers, and the aggregate effect and secondary reaction of fly ash [18]. By incorporating bentonite to regulate the impermeability of cement–soil walls, the study found that the minimum permeability coefficient of slag cement–bentonite material can reach 10^−18^ cm/s; furthermore, the addition of bentonite can reduce the permeability coefficient of cement–bentonite consolidations to below 10^−17^ cm/s at 28 days of curing age [19,20,21]. Using fly ash to replace part of the cement material as grouting material, conducting a feasibility study analyzing its impact on cement compressive strength [22,23,24]. In a study on the mechanical and hydraulic properties of cement–soil composites reinforced with sisal fiber and fly ash, it was found that the combination of fiber and fly ash significantly improved the peak strength of the cement–soil. Meanwhile, the fiber reduced permeability, whereas the fly ash increased it [25]. In terms of modifying cemented soil materials, existing studies mostly focus on the mechanism of action of single admixtures, which does not align well with complex working conditions.

While previous research has individually demonstrated the structural enhancement potential of the CSM method and the respective contributions of fiber, bentonite, and fly ash to cemented soil performance, no study has yet addressed the collaborative design and systematic optimization of an integrated system combining the CSM–H–section steel support with a basalt fiber–sodium bentonite–fly ash multi–component cementitious material, particularly under the complex geological conditions characteristic of highly weathered strata. Therefore, this paper proposes a CSM structure combined with H–beams, using ordinary Portland cement as the base matrix, adding basalt fiber, sodium bentonite, and fly ash to study the influence rules of each component on the new material, optimize the mix proportion for solidified cement–soil material, and compare the superiority of CSM with H–beams against bored piles through field engineering tests. It aims to solve the issues of economy, construction efficiency, and the common problems of insufficient solidified strength and poor impermeability of the formed cement–soil walls during construction, providing a practical solution for the efficient and economical application of the CSM method in complex strata.

## 2. Numerical Simulation

In conventional foundation pit support design, the use of CSM bored piles combined with anchor cables can effectively ensure stability during excavation. However, such temporary support structures are permanently buried upon completion of the foundation pit construction, resulting in neither recyclability nor significant wastage of construction materials, which contradicts the principles of green construction and sustainable development. To address this issue, this study proposes a more economical and environmentally friendly optimization scheme: leveraging the excellent waterproofing performance of the CSM method while replacing traditional bored piles with high–strength recyclable steel sections as the primary load–bearing components. This is combined with a detachable anchor cable system to form a composite support system. This approach not only meets stability requirements during excavation but also enables the recovery of key components such as steel sections and anchor cables upon project completion, facilitating material recycling. This innovative design maintains the reliability of the original support system while significantly reducing engineering costs and minimizing construction waste, offering a more economical, environmentally friendly, and sustainable support solution for foundation pit engineering.

Numerical simulations were conducted using two software packages: COMSOL Multiphysics (Version 6.0, developed by COMSOL AB, Stockholm, Sweden) for the simplified foundation pit model, and Lizheng Deep Foundation Pit Support Structure Design Software (version 7.5, developed by Beijing Lizheng Software Co., Ltd., Beijing, China) for the entire foundation pit.

### 2.1. Basic Support Parameters

**Figure 1:**
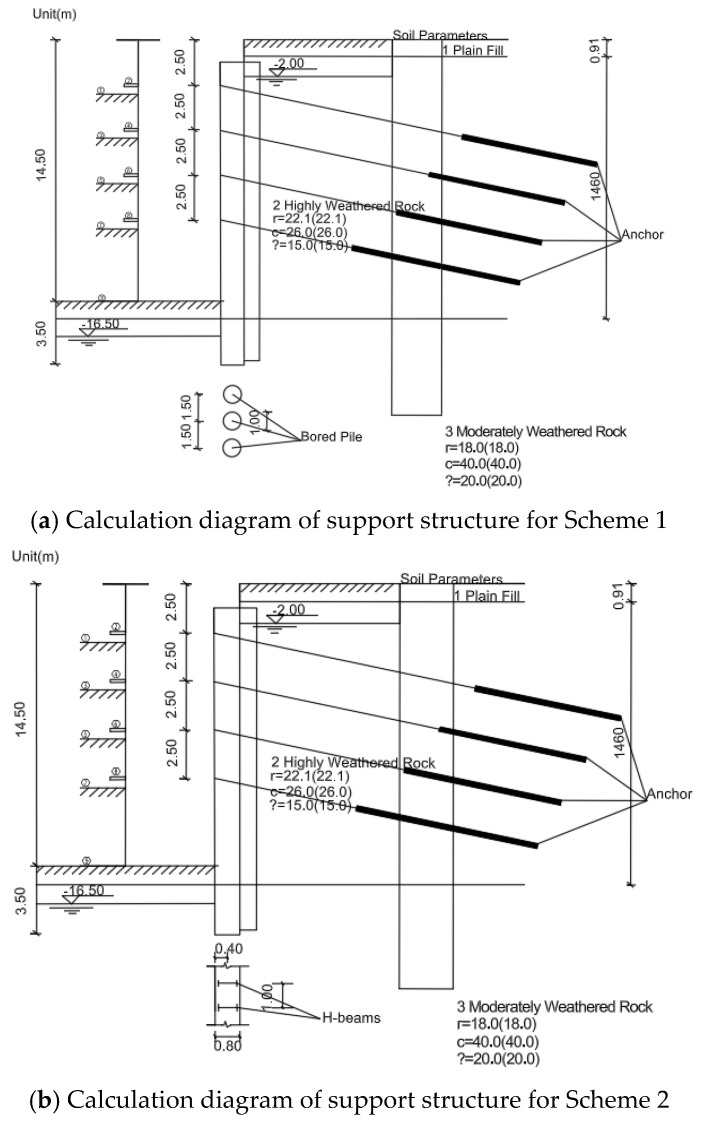
Schematics of support structures.

Scheme 1: The foundation pit is supported using bored piles combined with anchor cables and a CSM waterproof curtain. The support schematic is shown in Figure 1a. The embedded depth is 3.5 m, the pile cross–section is circular with a diameter of 1000 mm, and the pile spacing is 1500 mm. The waterproof curtain consists of a 700 mm thick CSM wall, with vertical anchor cables for additional support. The bottom of the CSM waterproof curtain should extend into the weakly permeable rock layer by no less than 1.2 m.

Scheme 2: The foundation pit is supported using the CSM method with embedded H–section steel piles combined with anchor cables. The support schematic is shown in Figure 1b. The CSM wall thickness is 800 mm, and the embedded H–section steel model is HN500 × 200. The steel piles are arranged at equal intervals of 1.0 m, with an embedded depth of 3.5 m. Vertical anchor cables are also installed for additional support.

COMSOL Multiphysics was selected for its suitability for multi–physics coupling, enabling effective simulation of soil–structure interaction. The soil was modeled using the ideal elastic–plastic Mohr–Coulomb model. The boundary conditions were set as follows: the bottom of the model was fixed, the lateral boundaries were constrained in the normal direction, and the top boundary was free.

The simulation primarily analyzed ① horizontal displacement of the support structure (maximum displacement and its location); ② bending moment of the wall/pile; ③ axial force of the struts; ④ overall stability safety factor.

### 2.2. Basic Information

**Figure 2:**
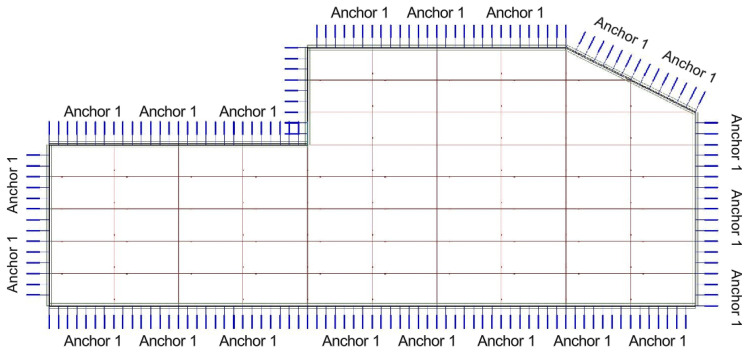
Plan layout of internal bracing system.

The computational model can be simplified as follows: The pit length is 100 m, the width is 25 m, and the excavation depth is 14.5 m. At this support section, four layers of steel waling beams are arranged in a hinged configuration, and four rows of anchor cables are considered in the calculation. Based on the COMSOL Multiphysics software, a comparative study is conducted between the original support scheme and the support scheme using the new CSM construction method. The internal forces in the engineering piles and walls, as well as the displacements of the piles and walls, are compared. A plan view of the internal bracing levels is shown in Figure 2:

The entire foundation pit is reinforced with anchor cables. The anchor cable specifications are detailed in Table 1, and the soil layer properties were determined based on the laboratory soil test results from the detailed investigation report of the Qingdao Metro Haixi Road Station project, as presented in Table 2. The over–excavation depth is 0.5 m. The calculation working conditions are summarized in Table 3.

### 2.3. Calculation Results

(1) Global Displacement

**Figure 3:**
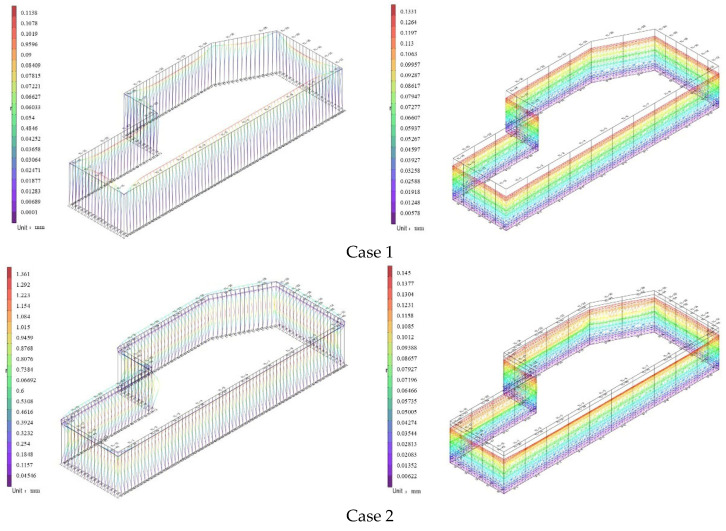

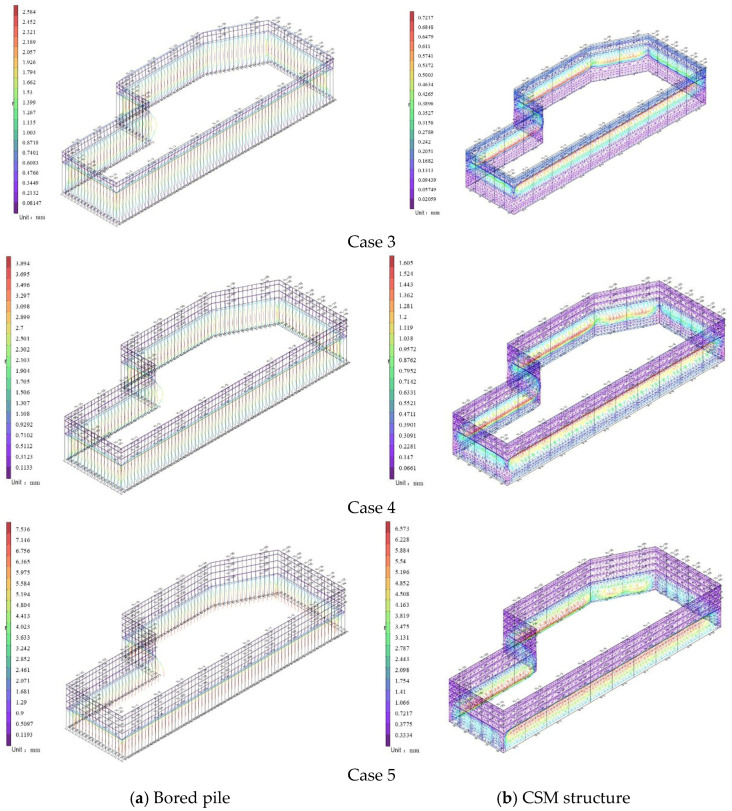
Variation in global displacement for the two support structures.

As shown in Figure 3, the variation process of the global displacement of the two support structures during the foundation pit excavation is illustrated.

In Case 1, due to the absence of any constraints and the relatively shallow excavation depth, the displacement of the support structure was most pronounced at the top of the piles and walls, with horizontal displacement directed toward the interior of the foundation pit. As excavation proceeded further, both types of foundation pit support exhibited rigid support behavior; consequently, the wall displacement manifested as triangular horizontal displacement. This differs from flexible walls, which, after the installation of struts or anchor cables, typically show unchanged displacement at the wall top or movement toward the exterior of the pit. As indicated by Case 3, the wall abdomen bulged toward the interior of the pit. By the time excavation reached the pit bottom (Case 5), the inward displacement of the walls or piles gradually decreased from the bottom upward. The variation characteristics of wall displacement during excavation generally conform to the typical patterns observed in foundation pit excavation.

Additionally, due to the release of self–weight stress during soil excavation, the piles and walls exhibited a tendency to heave upward. This upward movement negatively impacts pit stability, surface settlement safety, and the effectiveness of the support structure. This phenomenon was reflected in the pile and wall deformations calculated by Lizheng 7.5, although the changes were not pronounced.

(2) Internal Forces in Retaining Structures

**Figure 4:**
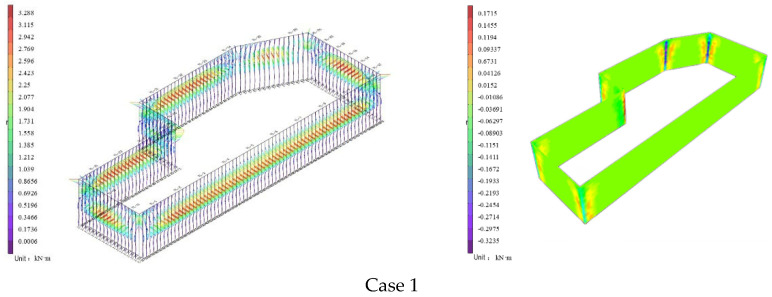

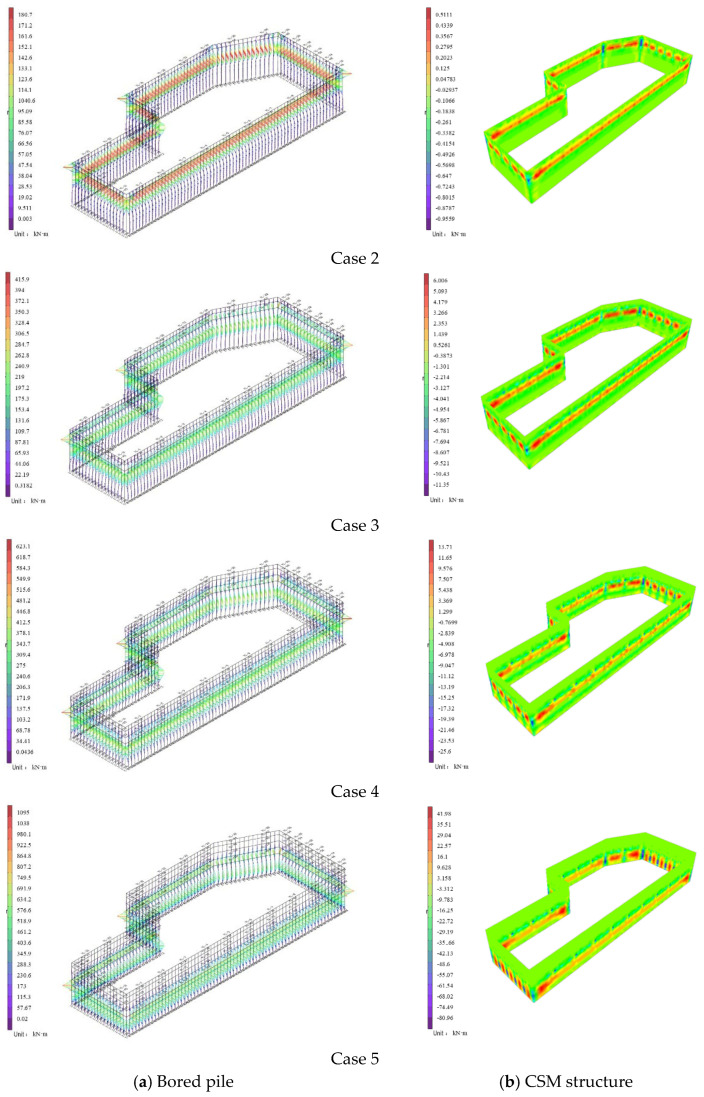
Internal forces in piles and walls during excavation process.

Figure 4 illustrates the development process of internal forces in piles and walls during foundation pit excavation, based on theoretically calculated earth pressure distributions at different depths, and considering the effects of hydrostatic pressure and seepage caused by groundwater level changes on piles and walls.

In the bored pile support structure, as excavation progresses, the location of the maximum bending moment of the pile moves toward the pile bottom. The maximum bending moment on the excavation side is 423.95 kN·m, located near the pile bottom, indicating that earth pressure is concentrated in the deep zone of the retaining side. The maximum bending moment on the retaining side is −653.1 kN·m, directed toward the exterior of the foundation pit, caused by soil rebound after excavation unloading and the constraint effect of the foundation base. As excavation depth increases, the location of the extreme bending moment shifts from the pile top to the pile bottom, reflecting the gradual mobilization of the passive zone soil constraint.

The maximum shear force and axial force also appear at the location of the maximum bending moment, with values of 494.77 kN. At the maximum bending moment on the excavation side, the axial force is 222.29 kN, while on the retaining side it is 155.27 kN, possibly due to uneven distribution of earth pressure on both sides or asymmetric reinforcement of the support structure. The migration of the bending moment location is attributed to the staged excavation effect: in the initial stage, the pile top is free, and the extreme bending moment is located at the pile top; as excavation deepens, the passive resistance of the foundation soil increases, constraining pile bottom displacement, and the extreme bending moment moves downward. The higher modulus of deep soil layers limits pile deformation, exacerbating the bending moment at the pile bottom.

The internal force distribution of foundation pit support piles is jointly influenced by earth pressure, water level changes, and construction sequence. The concentration of bending moment in the deep zone of the retaining side requires optimization through increased pile diameter, reinforcement, and collaborative support. During dynamic construction, the support scheme should be adjusted in real–time based on monitoring data to ensure structural safety.

In the CSM method support structure, as the support process progresses, the region of maximum wall bending moment divides the entire wall into three parts: the upper stabilized support zone, the middle stress zone, and the lower unexcavated zone. The location of the maximum bending moment of the wall moves toward the wall bottom. The vertical bending moment is 88.88 kN·m on the excavation side and −166.93 kN·m on the retaining side. The former is mainly caused by active earth pressure, while the latter reflects the constraint effect of the passive zone soil and the concentration of deep earth pressure. The horizontal bending moment is 37.83 kN·m on the excavation side and −64.10 kN·m on the retaining side, indicating the presence of asymmetric loads in the horizontal direction, which arise due to the asymmetric form of the foundation pit. The vertical shear force is 32.45 kN, and the horizontal shear force is 54.47 kN.

During foundation pit excavation, the internal force distribution of the CSM method support structure is jointly influenced by earth pressure, water level, and synergistic effects. The concentration of vertical bending moment on the retaining side requires optimization by improving interfacial shear stiffness and increasing support density. Although the horizontal bending moment is relatively small, attention should be paid to the influence of asymmetric loads. During construction, the support scheme should be adjusted in real–time based on monitoring data to ensure foundation pit stability.

Through the above comparison, the CSM support structure demonstrates good adaptability in design and construction. Future research could further study the mechanism of interface interaction to enhance engineering reliability under complex geological conditions.

(3) Internal Forces (Axial Force) in Top Beam or Waling Beams

**Figure 5:**
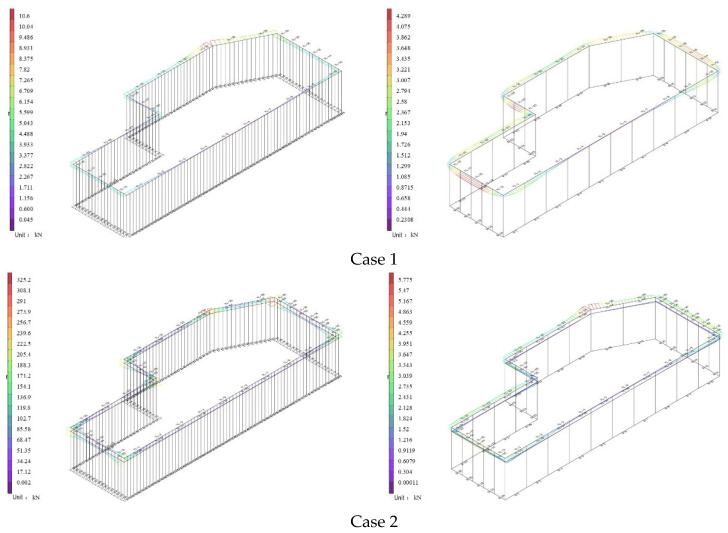

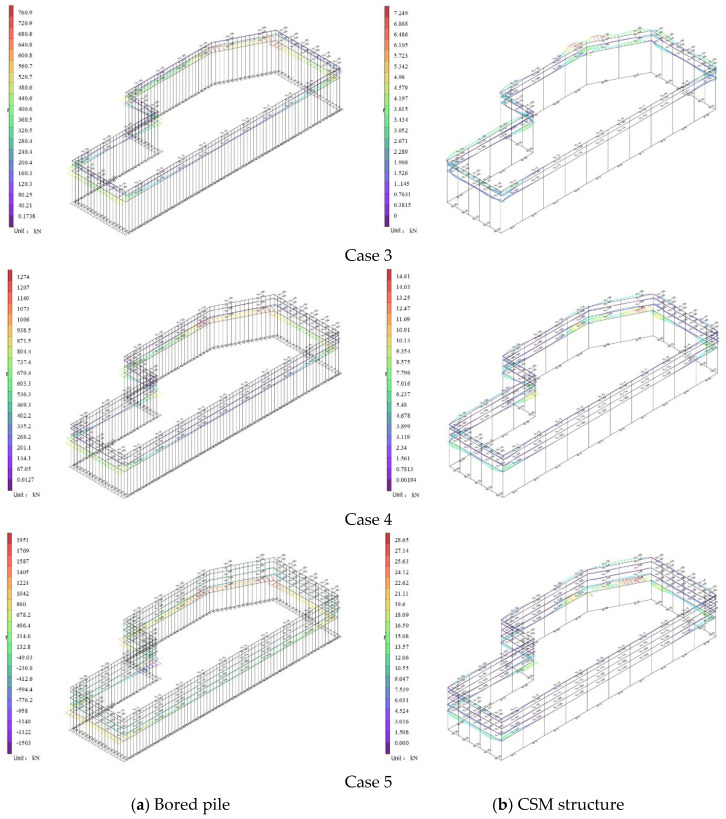
Magnitude of axial force in waling beams for the two support structures.

As horizontal support members, the top beam or waling primarily bears the horizontal loads transmitted from earth pressure to the retaining piles or diaphragm walls and transfers these loads to the opposing support structures or anchor systems through its axial stiffness. Figure 5 shows the axial force of the waling when the retaining structure is excavated to the pit bottom; the magnitude of this axial force directly reflects the level of earth pressure sustained by the support system.

Figure 5a presents the axial force in the waist beam of the bored pile support system upon excavation to the pit bottom. For waist beams with large spans, bending deformation may occur due to non–uniform earth pressure distribution, construction–induced eccentric loads, or support spacing, while insufficient joint stiffness can induce additional moments. Taking waist beam YL–50 as an example (with compression taken as positive), the maximum axial force is 19.51 kN, indicating that the waist beam is in compression and resists horizontal earth pressure. The relatively low axial force may be attributed to: (1) uniform load sharing within the support system, with the waist beam bearing only localized earth pressure; (2) small support spacing or high beam stiffness leading to load dispersion; or (3) the absence of soil nonlinearity or seepage coupling in the numerical model, potentially underestimating the axial force. The axial force distribution in the piles follows a trend consistent with that in the waist beam (higher at the pile top and lower in the waist beam), confirming effective load transfer and a collaborative load–bearing mechanism.

Figure 5b shows the axial force in the waist beam of the CSM support system at the pit bottom. The maximum axial force reaches 28.65 kN at the wall corner, and the internal force distribution in the wall aligns with the axial force trend in the waist beam, indicating effective load transfer and a collaborative system. Anchor bolts carry the majority of the load, while the waist beam acts as a secondary load transfer path, resulting in lower axial forces. In practice, construction monitoring should be enhanced by installing vibrating–wire axial force gauges at critical sections of the waist beam to track axial force variations in real time and validate design assumptions.

Through the above comparison, both the bored pile and CSM support structures meet safety requirements under the current working conditions. Future designs could further optimize the load distribution mechanism to enhance the economy and reliability of the support system.

## 3. Materials Proportioning Study

To address the engineering bottlenecks associated with the CSM (Cutter Soil Mixing) method in highly weathered strata—specifically, the low unconfined compressive strength and high permeability coefficient of the solidified cement soil—this study is based on the principle of material composite modification. Using ordinary Portland cement as the base material, a multi–component synergistic reinforcement system was constructed by incorporating admixtures such as basalt fiber, sodium bentonite, and fly ash. By comparing the influence patterns of different mix proportions, the optimal material combination was determined. The development of this new composite curing agent provides an innovative material solution for improving the quality of CSM diaphragm walls in highly weathered strata

In concrete, basalt fiber functions as a reinforcing material through micro–reinforcement and crack resistance mechanisms. Fly ash serves as a supplementary cementitious material that predominantly affects later–age strength, durability, and workability. Sodium bentonite is employed as a rheology modifier or waterproofing admixture to control the fluidity of the concrete mixture.

### 3.1. Raw Materials

(1) Base Material

The cement used in the test was Grade 42.5 ordinary Portland cement produced by Henan Mengdian Group Cement Co., Ltd. (Xinxiang, China). The performance indicators of the cement are shown in Table 4.

(2) Fiber

The basalt fiber used in the test was chopped fiber produced by Shanghai Chenqi Chemical Technology Co., Ltd. (Shanghai, China), with a specification of 12 mm. Its specific performance is shown in Table 5.

(3) Additives

The composition of the sodium bentonite used in the test is shown in Table 6.

Some physics and chemical parameters of fly ash used in the test are shown in Table 7.

### 3.2. Test Scheme

Based on the findings of preliminary tests, the following material proportions were adopted: basalt fiber volume fraction of 0.3–0.5%, fly ash replacement rate of 5–15%, and sodium bentonite content ranging from 3% to 5%—the latter selected to achieve an optimal balance between impermeability and strength loss.

This test adopted the water–cement ratio of 1.0 required by the background project design. The basalt fiber specification was 12 mm with incorporation rates of 0.3%, 0.4%, and 0.5%. Fly ash replaced ordinary Portland cement at the same mass fractions of 5%, 10%, and 15%. Sodium bentonite incorporation rates were set at 3%, 4%, and 5%, respectively. Specimens at ages of 3 d, 7 d, and 28 d were selected as variables. A total of 37 different test conditions were set up. The specific material mix proportions are shown in Table 8. Multiple groups of mixed ratio–related tests were configured for the solidified cement material. The fluidity, bleeding rate, setting time, and other properties of the material’s neat paste slurry were tested and recorded, and the results were analyzed. Using relevant test instruments, the compressive strength and flexural strength of the consolidated solid for the solidified cement material under different conditions were tested. The test data were organized and analyzed, and the final results were compared to summarize the influence of material mix proportions on various properties of the solidified cement material under different conditions, ultimately determining the optimal mix proportion for the solidified cement material. A four–factor, three–level orthogonal experiment (L(3^4^)) was established, with the four factors determined as: basalt fiber (A), fly ash (B), sodium bentonite (C), and a blank column for error (D), orthogonal test scheme is shown in Table 9. Three levels were chosen for each factor. Based on the aforementioned test data results, basalt fiber mass fractions of 0.3%, 0.4%, and 0.5% were selected; fly ash levels of 5%, 10%, and 15% were selected; sodium bentonite levels of 3%, 4%, and 5% were selected.

### 3.3. Research Result

This study prepared 37 groups of solidified cement paste slurries with different mix proportions, including two control groups with water–cement ratios of 1.0 and 0.5, D1.0 and D0.5, which were plain cement paste without any additives. The rest were test groups for solidified cement materials with different mix proportions, details are shown in Table 10.

#### 3.3.1. Setting Time Analysis

The setting time of the solidified cement slurry was determined using the Vicat apparatus, following the procedures specified in the Chinese national standard Test methods for water requirement of normal consistency, setting time and soundness of the Portland cement (GB/T 1346) [26].

Setting time serves as a critical parameter for solidified cement slurry materials. An excessively short setting time prevents adequate mixing between the slurry and in situ soil, resulting in non–uniform wall strength distribution and compromised construction quality. Conversely, an overly prolonged setting time promotes slurry segregation, leading to excessive water permeation and increased construction hazards. Consequently, the setting time of solidified cement slurry materials should be designed to be controllable within a specified range while adhering to relevant specifications. The orthogonal experimental design and range analysis for initial setting time and final setting time are shown in Table 11 and Table 12.

Based on the range analysis, the influence on the initial setting time follows the order: fly ash > sodium bentonite > basalt fiber, as indicated by the mean values at each level. The optimal combination (A_2_B_3_C_1_) was determined by selecting the level corresponding to the maximum value of k_i_ was selected for each factor. The initial setting time of the slurry is significantly affected by the material proportions, with fly ash content and sodium bentonite content being the predominant influencing factors.

The primary reactants in cement hydration are cement clinker and water. During the induction period, the addition of fly ash and sodium bentonite partially impedes contact between cement and water, leading to a slower initial hydration rate. In the acceleration period, a hydration product layer forms on the cement surface. The SiO_2_ and Al_2_O_3_ components in fly ash undergo a secondary hydration reaction with Ca(OH)_2_—a product of cement hydration—generating calcium silicate hydrate (C–S–H) and other phases that contribute to water retention. As interparticle spacing increases, the time required for hydration products to fill the skeletal pores during setting is prolonged, thereby extending the overall setting time.

In summary, based on the final setting time, the optimal mix design is determined to be A_3_B_3_C_1_, corresponding to a basalt fiber content of 0.5%, fly ash content of 15%, and sodium bentonite content of 3%.

#### 3.3.2. Bleeding Rate Analysis

Basalt fiber has a special spatial network structure, giving it good physical properties including electrical insulation and dielectric properties [27]. These properties allow basalt fiber to form a uniform three–dimensional randomly distributed network system in the cement paste, which can slow down water evaporation and migration, thus affecting the bleeding rate of the cement paste. Sodium bentonite has good adsorption and high cation exchange capacity; it can adsorb and inhibit the sedimentation of cement particles, thereby reducing the bleeding rate of the slurry [28]. The main chemical components of fly ash include SiO_2_, Al_2_O_3_, and Fe_2_O_3_, accounting for over 70% of its total content. These active materials, when mixed with cement, participate in the hydration reaction, thus affecting the stability of the slurry and consequently the bleeding rate of the cement paste.

The degree of influence on slurry bleeding rate is fly ash > sodium bentonite > basalt fiber. Therefore, the material mix proportion A_1_B_1_C_1_ has the highest bleeding rate. Considering bleeding rate, the scheme with the highest bleeding rate is A_1_B_1_C_1_; the scheme with the lowest bleeding rate is A_3_B_3_C_3_. Further comprehensively considering other properties of the cement paste, the better combination is A_3_B_3_C_1_, i.e., basalt fiber content 0.5%, fly ash content 15%, sodium bentonite content 3%.

**Figure 6:**
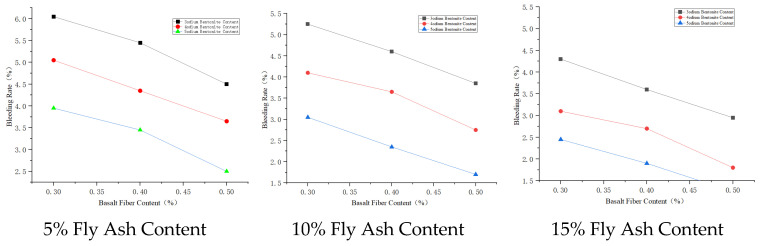
Effects of basalt fiber on bleeding rate.

**Figure 7:**
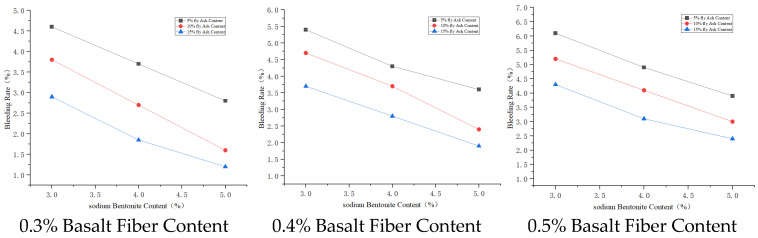
Effects of sodium bentonite on bleeding rate.

**Figure 8:**
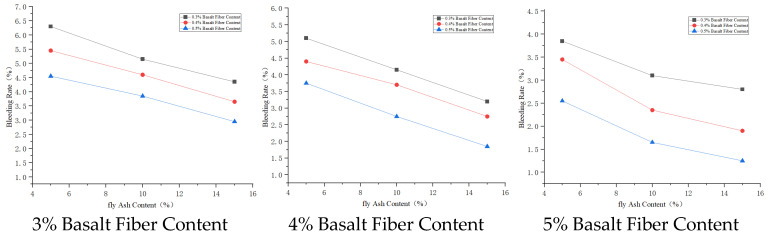
Effects of fly ash on bleeding rate.

The effect of admixture dosage on bleeding rate is shown in Figure 6, Figure 7 and Figure 8.

#### 3.3.3. Fluidity Analysis

The fluidity of the solidified cement slurry material was tested using the following apparatus: a cement paste mixer, a truncated cone mold (upper diameter: 36 mm, lower diameter: 60 mm), a glass plate (500 mm × 500 mm × 3 mm), and a steel ruler.

Basalt fiber has stable chemical properties, remaining stable in the slurry, not easily hydrolyzed or chemically decomposed, thus reducing the fluidity of the cement paste. Sodium bentonite, due to its expansibility and adsorbability, forms a uniform three–dimensional randomly distributed network system in the cement paste, which can slow down water evaporation and migration. Due to the particle size and surface positive charge characteristics of cement particles, montmorillonite particles are attached to the surface of relatively larger cement particles under electrostatic attraction and van der Waals forces, reducing the mobility of cement particles and thus decreasing slurry fluidity. Slurry fluidity increases with the increase in fly ash content, and the rate of increase gradually becomes larger. When fly ash particle fineness is less than that of cement particles, it can fill the internal pores of cement particles, enhancing paste fluidity; when the number of glass microbeads in fly ash increases, the microbeads can reduce the slip resistance between cement particles, significantly improving slurry fluidity.

The fly ash content has a significant effect on slurry fluidity. Within the 5~15% content range, the content is positively correlated with slurry fluidity. The influence on slurry fluidity is fly ash content > sodium bentonite content > basalt fiber content. The reason is that the silicon dioxide (SiO_2_) and aluminum oxide (Al_2_O_3_) components in fly ash can undergo secondary hydration reaction with calcium hydroxide (Ca(OH)_2_) generated during cement hydration. The resulting substances like calcium silicate hydrate have water–retaining functions, not only enhancing the lubricating effect of the ultra–fine cement paste but also achieving excellent fluidity while saving raw materials. For diaphragm wall engineering, which requires high fluidity, the mix proportion A_2_B_3_C_1_ is appropriate for the solidified cement material.

While macroscopic mechanical and permeability tests confirm that the combined addition of basalt fiber, fly ash, and bentonite substantially enhances the physical and mechanical properties of cemented soil, this study did not employ microscopic characterization techniques such as scanning electron microscopy (SEM) or mercury intrusion porosimetry (MIP) to directly observe the microstructural evolution. Drawing on findings from the literature, the observed performance improvements are likely attributable to: (1) the bridging and crack–resisting effect of basalt fibers through interfacial bonding with the cement matrix, which inhibits microcrack propagation under load; and (2) the combined pore–filling effect of C–S–H gel from the pozzolanic reaction of fly ash and the gel formed by bentonite upon water absorption and expansion, which reduces the connected porosity. Nevertheless, further microscopic investigation is needed to elucidate the fiber–clay mineral interactions within the interfacial transition zone and the specific influence of multi–component admixtures on the type and morphology of hydration products.

**Figure 9:**
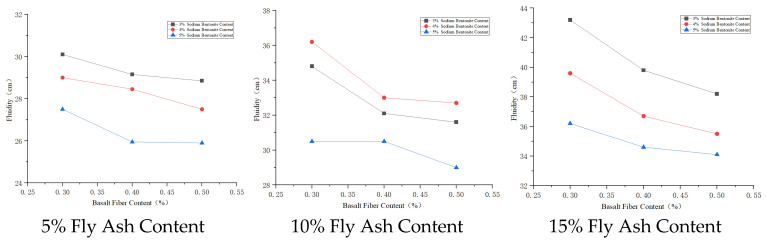
Effects of basalt fiber on fluidity.

**Figure 10:**
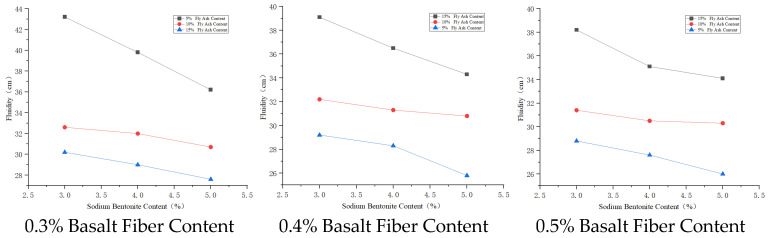
Effects of sodium bentonite on fluidity.

**Figure 11:**
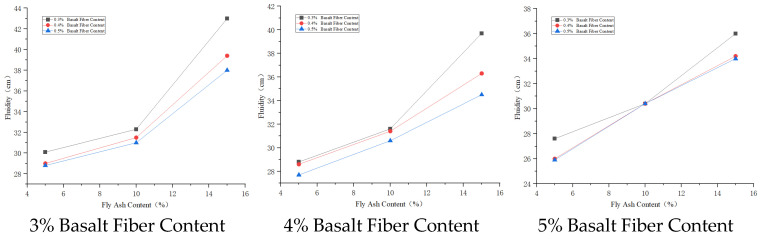
Effects of fly ash on fluidity.

The effect of admixture dosage on fluidity is shown in Figure 9, Figure 10 and Figure 11.

#### 3.3.4. Mechanical Strength Analysis

(1) Flexural Strength

The flexural strength was measured using a CDT1305–2 microcomputer–controlled electronic pressure testing machine via the three–point bending method. A displacement–controlled load was applied at a rate of 0.4 mm/min, and the ultimate load at failure was recorded.

For both tests, if one of three values deviated by more than ±10% from the mean, the mean of the remaining two values was taken; if two values deviated by more than ±10%, the remaining single value was adopted as the result.

Basalt fiber content was found to dominate the early flexural strength of neat paste specimens, with strength increasing as fiber content increased. The relative importance of the three admixtures changed with curing age: at 3 and 7 days, the order of influence was basalt fiber > sodium bentonite > fly ash; at 28 days, however, fly ash became the most significant factor, followed by basalt fiber and then sodium bentonite.

The addition of basalt fiber can improve the microstructure of the cement paste, enhance its crack resistance, thereby increasing flexural strength. However, excessive incorporation will lead to deterioration of fiber dispersion, creating stress concentration weak areas inside the concrete, causing decay in the fiber interface bond strength. The water absorption of sodium bentonite may affect the early strength development of the cement paste, consequently affecting flexural strength. The content of sodium bentonite needs to be determined reasonably to achieve the best balance of flexural strength performance. The addition of fly ash can improve the pore structure of the cement paste, making it denser, thus increasing flexural strength. Increasing fly ash content significantly enhances the 28 d flexural strength of the solidified cement paste, making it an effective reinforcing material. The range analysis of flexural strength is presented in Table 13.

(2) Compressive Strength

Compressive strength was determined using a CDT1305–2 microcomputer–controlled electronic pressure testing machine at a loading rate of 2400 N/s, following GB/T 17671 (ISO method). The water–to–cement ratio was 1.0. Tests were performed on the two halves of specimens obtained from the flexural test, and the average value was reported as the compressive strength.

The order of influence of each factor on the compressive strength of neat paste specimens varied with curing age. At 3 and 7 days, the sequence was basalt fiber > sodium bentonite > fly ash. At 28 days, however, the order shifted to fly ash > basalt fiber > sodium bentonite. Fly ash content significantly influenced later compressive strength; at 28 days, the hydration degree of fly ash was limited, and the specimens were not fully mature. The later strength can still increase by 20–30% at ages of 90 to 180 days.

The addition of basalt fiber can improve the ultimate compressive strength of the specimens. Basalt fiber forms a microskeleton structure inside the concrete, altering the pore structure, making the interior of the specimen denser, which is beneficial for increasing its strength. After bentonite is incorporated, hydration reactions form cementitious products, effectively filling micropores between soil particles and sealing gaps in the aggregate structure, thereby reducing the porosity of the cement paste system. When the bentonite content gradually increases, the filling effect of high incorporation (10%) bentonite plays a major role in improving compressive strength, rather than the cement skeleton [29]. As fly ash content increases, upon contact with Ca(OH)_2_, it hydrates, and the resulting C–S–H gel products act synergistically with the hydration products of cement clinker, the strength of the solidified cement specimen generally rises, but the increase amplitude is significantly affected by the fiber content. The range analysis of Compressive Strength is presented in Table 14.

Range analysis shows: Basalt fiber content dominates the early flexural strength of the paste specimen, and its content is positively correlated with strength. Basalt fibers are randomly distributed within the cement paste specimen. In the internal space of the specimen, due to their high elastic modulus, they act as distributed reinforcement linking the cement skeleton, bearing external loads. When bearing load, basalt fibers dissipate externally applied energy through mechanisms like fiber pull–out, fracture, and deformation, effectively enhancing the compressive and flexural strength of the concrete cube. The results indicate that basalt fiber primarily influences early strength, whereas fly ash governs later strength. Considering later compressive strength, the better combination for the slurry material mix proportion is A_3_B_3_C_1_.

#### 3.3.5. Solidification Test

The cement content was calibrated based on the stratum parameters to ensure that the wall strength meets the safety threshold for foundation pit excavation. The control value for the 28–day unconfined compressive strength was set at ≥0.8 MPa, and the critical cement mixing ratio was taken as 20%. The water–to–cement ratio was optimally selected within the range of 1.0–2.0, and the permeability coefficient was required not to exceed 10^−7^ cm/s.

Specimens for strength testing were prepared using 100 mm cube molds. Permeability test specimens were 50 mm cubes, with at least three specimens per group. All specimens were cured for 24 h in a curing chamber maintained at a temperature of 21 ± 5 °C and a relative humidity greater than 90%. The test–related content is presented in Table 15.

(1) Consolidated Solid Compressive Strength

The unconfined compressive strength of the solidified cement soil specimens was measured using a WD–96205 microcomputer–controlled electronic universal testing machine.

**Figure 12:**
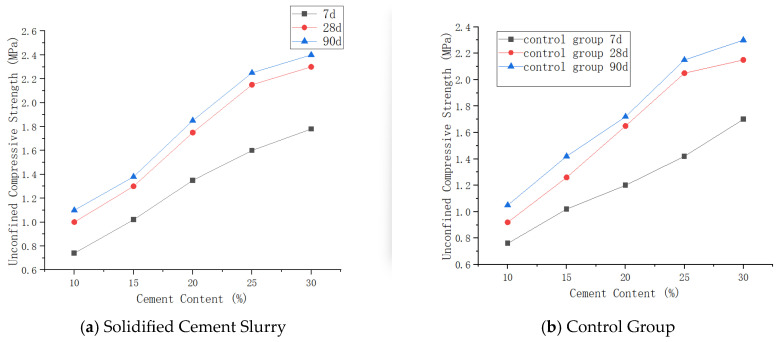
Curve of cement–soil consolidated solid strength versus cement content.

The unconfined compressive strength of the cement–soil consolidated solid increases with the increase in cement content. Compared to the control group, the new cement material can enhance the unconfined compressive strength of the cement–soil consolidated solid, and the effect becomes more obvious with increasing age. Through testing, cement–soil consolidated solids with a content above 15% can meet the quality assurance requirements for CSM wall formation. Based on practical engineering needs, a cement content of 20% is suitable for highly weathered stratum conditions. The variation curve of cemented soil strength with cement content is shown in Figure 12.

(2) Consolidated Solid Permeability Coefficient

The permeability coefficient, which reflects the impermeability of the cemented soil, was determined using a rock permeameter with the constant head method, following Darcy’s law.

**Figure 13:**
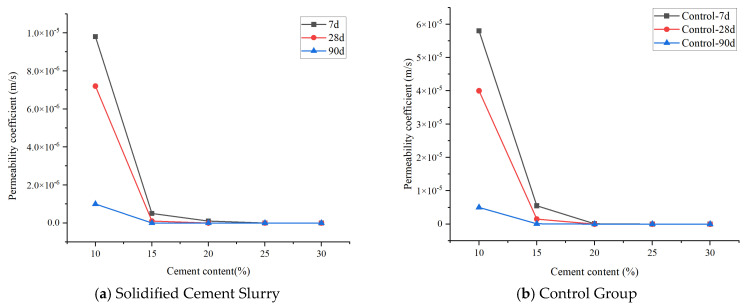
Curve of cement–soil consolidated solid permeability coefficient versus cement content.

The influence of solidified cement content on the permeability coefficient of the cement–soil consolidated solid shows that increasing content drives the permeability coefficient negatively correlated. When the content reaches the critical value of 10%, the permeability coefficient of the consolidated solid is significantly higher, unable to meet the seepage prevention threshold requirements for CSM engineering. When the cement content increases further, levels above 15% all meet the requirements. Compared to the control group, the cement content is reduced by 5%. The rate of decrease in permeability coefficient reduces as the cement content increases. Therefore, subsequently increasing cement content to reduce the permeability coefficient will significantly reduce economy. The variation curve of the permeability coefficient of cemented soil with cement content is shown in Figure 13.

Basalt fiber provides physical reinforcement through bridging and crack resistance mechanisms, effectively inhibiting the initiation and propagation of microcracks under load, thereby enhancing the toughness and post–peak load–bearing capacity of the matrix. Meanwhile, fly ash contributes chemical reinforcement through its pozzolanic effect, in which reactive SiO_2_ and Al_2_O_3_ consume calcium hydroxide to generate secondary C–S–H gel, densifying the microstructure. Its morphological effect improves workability, while the micro–aggregate effect further fills capillary pores. Sodium bentonite serves as a supplement through physical filling and waterproofing action: upon contact with water, its hydration–induced expansion characteristics can block macroscopic seepage channels, and its fine particles, combined with cation exchange capacity, fill interstitial pores and adsorb onto the surfaces of cement particles, significantly reducing permeability.

Crucially, these mechanisms do not operate in isolation but work synergistically. Fiber reinforcement compensates for the brittleness of the cementitious matrix, while the densifying effect of fly ash pozzolanic reaction provides a stronger interface for fiber anchoring, maximizing reinforcement efficiency. Simultaneously, the expansive filling action of bentonite, combined with the microstructural refinement by fly ash, creates a dual–scale sealing effect, significantly enhancing impermeability and long–term durability.

Ultimately, this ternary system constructs a three–dimensional modification network characterized by “fiber reinforcement–pozzolanic densification–bentonite sealing.” The resulting composite material exhibits simultaneous improvements in strength, toughness, and impermeability, with comprehensive performance far exceeding that achievable by any single additive alone.

## 4. Field Test

### 4.1. Project Overview

**Figure 14:**
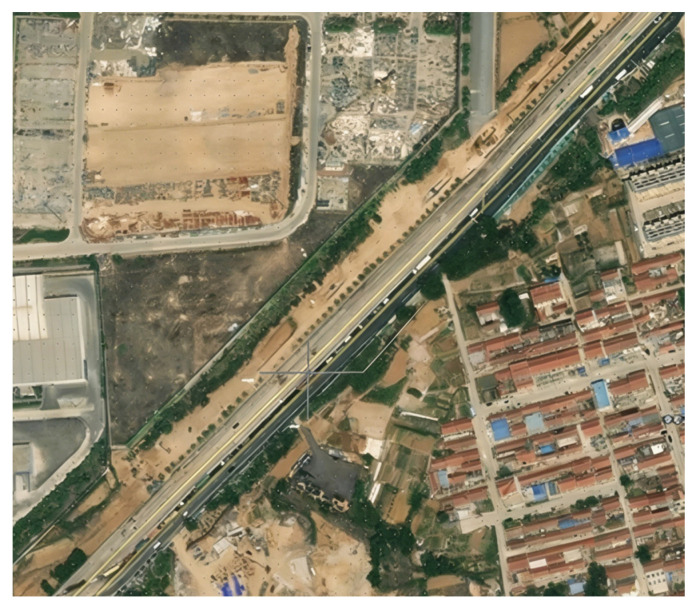
Satellite photo of Haixi Road Station.

Haixi Road Station is located between the intersection of Shuangfengshan Road and Haixi Road and the intersection of Shuangfengshan Road and Yujin Road, arranged perpendicular to Shuangfengshan Road from northwest to southeast. The satellite photo is shown in Figure 14. The geological structure pattern of this area is mainly controlled by the regional fault system, belonging to the secondary fault unit of the Cangkou Fault distributed in the NW direction, overall showing a NE extension characteristic. This fault has a strike of 134°, dipping southeast, with a dip angle of 70°, a plane extension length of about 14 km, and a structural fracture zone width of up to 30 m. The excavation is about 101 m long (longitudinal) and about 21.3~49.3 m wide (transverse), with a depth of about 22.4~27.9 m. The upper part of the excavation supporting structure uses the CSM method structural support, with an excavation depth of about 14.5 m. The lower part uses steel pipe piles + anchor cables, with an excavation depth of about 10.4 m. Locally, where the medium and slightly weathered rock surface is higher, a two–level steel pipe pile support type is used.

### 4.2. Engineering Effectiveness

**Figure 15:**
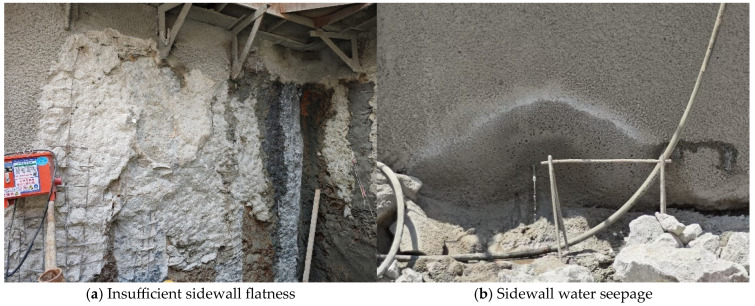
Common problems of bored pile support structure.

**Figure 16:**
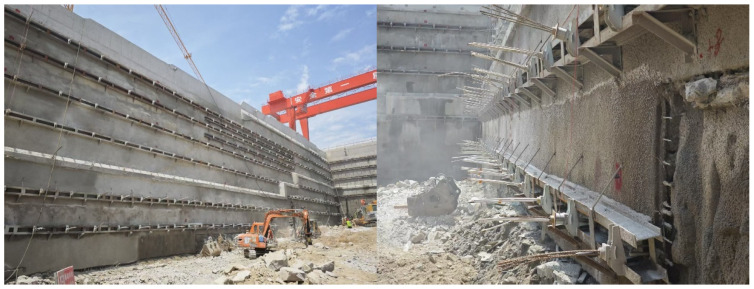
Support effect of CSM method structural support scheme.

During the actual construction process of the bored pile support structure scheme, due to various factors such as geological conditions and construction techniques, the excavation sidewall may exhibit issues like lack of flatness and water seepage, even noticeable water flow at a few locations. As shown in Figure 15. These problems not only affect the overall stability of the excavation but may also adversely impact subsequent construction and the safety of the structure. The support effect of the CSM method is shown in Figure 16.

The CSM method reduces cement usage, achieves a steel recovery rate of over 85%; shortens the construction period by 30%, reducing machinery rental and labor costs; reduces construction waste and carbon emissions, aligning with sustainable development requirements.

The CSM method structural support scheme uses a dual–wheel cutter device for in situ cutting and mixing of the stratum, coupled with a real–time verticality monitoring system (deviation ≤ 1/300), ensuring the wall is continuous, vertical, and uniform in thickness, forming a seamless continuous wall (permeability coefficient ≤ 1 × 10^−6^ cm/s), eliminating the seepage paths between piles inherent in bored pile support structures, thereby reducing the occurrence of the aforementioned problems. The comprehensive cost of the CSM method with embedded steel sections scheme is 19.1% lower than that of bored cast–in–place piles. The CSM method reduces cement usage, achieves a steel recovery rate of over 85%; shortens the construction period by 30%, reducing machinery rental and labor costs; reduces construction waste and carbon emissions, aligning with sustainable development requirements.

The cost of bored piles combined with waterproof curtain and CSM wall with embedded H–beams are shown in Table 16 and Table 17.

## 5. Conclusions

To address the technical challenges of low strength and poor impermeability of cemented soil walls when applying the CSM method in highly weathered strata, this study systematically optimized the CSM–H–section steel composite support structure and its associated cement–based material mix proportion through a combination of numerical simulation, orthogonal laboratory tests, and field validation. The main conclusions are as follows:

(1) The proposed CSM structure combined with H–section steel and detachable anchor cables demonstrates superior performance compared to traditional bored pile support systems. Numerical simulation results indicate that the optimized scheme achieves comparable displacement control while reducing the bending moment concentration at the wall toe. Field application at Haixi Road Station confirmed that this method effectively addresses the common issues of sidewall unevenness and water seepage associated with bored piles.
(2) Through orthogonal experiments and range analysis, the optimal mix proportion for the solidified cement–soil material in highly weathered strata was determined as: 0.5% basalt fiber (12 mm), 15% fly ash, and 3% sodium bentonite. This combination achieves the best balance between workability (fluidity, setting time, bleeding rate) and mechanical properties (compressive and flexural strength).
(3) The synergistic mechanism of the ternary additive system was elucidated: basalt fiber provides crack–bridging reinforcement, fly ash contributes pozzolanic densification, and sodium bentonite ensures physical sealing. This multi–level modification network simultaneously enhances strength, toughness, and impermeability, with the optimal cement content identified as 20% for engineering applications in the Huangdao area.
(4) Economic analysis reveals that the CSM method with embedded H–section steel reduces the comprehensive project cost by 19.1% compared to the traditional bored pile scheme, while achieving a steel recovery rate exceeding 85% and a 30% reduction in construction period, aligning with green construction and sustainable development principles.

Despite the achievements described above, this study has certain limitations: the laboratory tests were conducted under standard curing conditions, which cannot fully reflect the complex temperature and humidity variations encountered in the field; the long–term durability of the proposed material under wet–dry cycles and freeze–thaw cycles—common in weathered strata—has not yet been systematically evaluated; and the numerical simulation assumed a homogeneous stratum without considering the effects of construction disturbances or localized geological anomalies.

It is recommended that future research focus on long–term field monitoring, durability assessment, microstructural characterization, and optimization of construction parameters.

## Figures and Tables

**Table 1:** Anchor Cable Parameters of Sectional Support Units.

Anchor Cable ID	Tendon Type	Steel Waling Beam Model	Horizontal Spacing (m)	Inclination Angle (°)	Borehole Diameter (mm)	Effective Length (m)	Free Length (m)	Bond Length (m)	Prestress Lock–Off Value (kN)
Anchor–1	4Φs15.2	2I28b	1.5	15	150	17	10	7	300
Anchor–2	4Φs15.2	2I28b	1.5	15	150	16	9	7	400
Anchor–3	4Φs15.2	2I28b	1.5	15	150	16	8	8	400
Anchor–4	4Φs15.2	2I28b	1.5	15	150	14	7	7	400

4Φs15.2: 4 indicates the number of strands, Φ indicates the diameter symbol, and s15.2 indicates the diameter specification. 2I28b: 2 indicates the number of steel sections, I indicates I–shaped steel, and 28b indicates the model designation of the I–section steel.

**Table 2:** Soil Layer Information for Sectional Support Units.

Layer No.	Name	Thickness (m)	Unit Weight (kN/m^3^)	Buoyant Unit Weight (kN/m^3^)	Cohesion (kPa)	Internal Friction Angle (°)	Friction Resistance with Anchor (kPa)	Analysis Method	Total Stress Analysis
1	Plain Fill	0.91	17.9	–	14	18	30	M–Method	Yes
2	Highly Weathered Rock	14.60	22.1	12.1	26	15	300	M–Method	Yes
3	Moderately Weathered Rock	50.00	18.0	8.0	40	20	360	M–Method	Yes

**Table 3:** Calculation Working Conditions.

Working Condition No.	Excavation Depth (m)	Over–Excavation Depth (m)
1	−3.3	0.5
2	−5.8	0.5
3	−8.3	0.5
4	−10.8	0.5
5	Pit Bottom	0.5

**Table 4:** Performance Indicators of Ordinary Portland Cement.

Specific Surface Area/m^2^·kg^−1^	Setting Time (min)		Flexural Strength (MPa)		Compressive Strength (MPa)	
	Initial Set	Final Set	3 d	28 d	3 d	28 d
≥300	≥45	≤600	≥4.0	≥6.5	≥17.0	≥42.5
355	202	263	5.5	8.2	26.6	50.4

**Table 5:** Main Performance Parameters of Basalt Fiber.

Name	Specific Gravity	Tensile Strength	Elastic Modulus	Size	Moisture Content	Elongation at Break
Testing Standard for Basalt Fiber	2.8–3.3 g/cm^3^	≥1250 MPa	≥40 GPa	12 mm	≤0.2%	≥3.1%
Measured	2.86 g/cm^3^	2246 MPa	55 GPa	12 mm	0.1%	3.15%

**Table 6:** Chemical composition of the Sodium Bentonite.

Type	SiO_2_/%	Al_2_O_3_/%	Na_2_O/%	MgO/%	CaO/%	Fe_2_O_3_/%	Ti_2_O_3_/%
Content	64.59	17.23	3.26	2.21	1.28	2.65	0.36

**Table 7:** Some physics and chemical parameters of Fly Ash.

Standard	Loss on Ignition/%	Fineness/%	Moisture Content/%	Water Demand/%	SO_3_/%
National Standard	≤5.0	≤12	≤1.0	≤95.0	≤3.0
Measured Data	3.05	10.1	0.34	90	0.26

**Table 8:** Cementitious materials mix proportion.

No.	Test ID	Basalt Fiber Content	Sodium Bentonite Content	Fly Ash Content
1	BSF–1–1	0.3%	3%	5%
2	BSF–1–2	0.3%	4%	5%
3	BSF–1–3	0.3%	5%	5%
4	BSF–1–4	0.3%	3%	10%
5	BSF–1–5	0.3%	4%	10%
6	BSF–1–6	0.3%	5%	10%
7	BSF–1–7	0.3%	3%	15%
8	BSF–1–8	0.3%	4%	15%
9	BSF–1–9	0.3%	5%	15%
10	BSF–2–1	0.4%	3%	5%
11	BSF–2–2	0.4%	4%	5%
12	BSF–2–3	0.4%	5%	5%
13	BSF–2–4	04%	3%	10%
14	BSF–2–5	0.4%	4%	10%
15	BSF–2–6	0.4%	5%	10%
16	BSF–2–7	0.4%	3%	15%
17	BSF–2–8	0.4%	4%	15%
18	BSF–2–9	0.4%	5%	15%
19	BSF–3–1	0.5%	3%	5%
20	BSF–3–2	0.5%	4%	5%
21	BSF–3–3	0.5%	5%	5%
22	BSF–3–4	0.5%	3%	10%
23	BSF–3–5	0.5%	4%	10%
24	BSF–3–6	0.5%	5%	10%
25	BSF–3–7	0.5%	3%	15%
26	BSF–3–8	0.5%	4%	15%
27	BSF–3–9	0.5%	5%	15%
28	D–0	0%	0%	0%
29	B–1	0.3%	0%	0%
30	B–2	0.4%	0%	0%
31	B–3	0.5%	0%	0%
32	S–1	0%	3%	0%
33	S–2	0%	4%	0%
34	S–3	0%	5%	0%
35	F–1	0%	0%	5%
36	F–2	0%	0%	10%
37	F–3	0%	0%	15%

**Table 9:** Orthogonal Test Scheme.

Test ID	A (Basalt Fiber Level)	B (Fly Ash Level)	C (Bentonite Level)	D (Error)
1	1	1	1	1
2	1	2	2	2
3	1	3	3	3
4	2	1	2	3
5	2	2	3	1
6	2	3	1	2
7	3	1	3	2
8	3	2	1	3
9	3	3	2	1

**Table 10:** Experimental Results.

Test ID	Initial Set (min)	Final Set (min)	Bleeding Rate (%)	Fluidity (mm)	Compressive Strength (MPa)			Flexural Strength (MPa)		
					3 d	7 d	28 d	3 d	7 d	28 d
BSF–1–1	208	264	6.1	30.1	3.95	6.90	8.54	1.04	1.64	1.96
BSF–1–2	200	240	5.0	28.9	3.84	6.63	7.79	0.96	1.54	1.88
BSF–1–3	196	224	3.9	27.5	3.80	6.11	7.78	0.76	1.46	1.84
BSF–1–4	216	288	5.2	32.5	3.86	7.08	10.18	0.99	1.60	2.48
BSF–1–5	210	276	4.1	31.6	3.82	6.85	10.09	0.85	1.49	2.40
BSF–1–6	204	260	3.0	30.5	3.55	6.23	9.78	0.76	1.45	2.23
BSF–1–7	224	314	4.3	43.0	3.84	7.18	9.46	0.96	1.58	3.09
BSF–1–8	218	286	3.1	39.5	3.77	6.91	9.06	0.76	1.46	3.02
BSF–1–9	210	266	2.4	36.2	3.24	6.70	8.79	0.72	1.27	2.95
BSF–2–1	208	270	5.4	29.1	4.51	7.40	9.23	1.20	1.85	2.24
BSF–2–2	202	248	4.3	28.4	4.28	7.29	9.04	1.13	1.77	2.17
BSF–2–3	196	224	3.4	25.9	4.19	7.20	8.73	1.07	1.75	2.13
BSF–2–4	216	282	4.6	32.1	4.48	7.68	11.44	1.25	1.79	2.98
BSF–2–5	208	276	3.6	31.2	4.21	7.38	11.10	1.17	1.76	2.93
BSF–2–6	204	264	2.3	30.5	4.14	7.23	10.89	1.07	1.65	2.75
BSF–2–7	224	314	3.6	39.5	4.28	7.73	10.15	1.13	1.78	3.50
BSF–2–8	216	284	2.7	36.5	4.20	7.39	10.08	1.08	1.75	3.32
BSF–2–9	210	264	1.9	34.3	4.10	7.30	9.60	1.06	1.65	3.17
BSF–3–1	208	268	4.5	28.8	6.39	9.00	10.13	1.96	2.93	2.75
BSF–3–2	200	242	3.6	27.5	6.12	8.29	9.92	1.47	2.37	2.75
BSF–3–3	196	218	2.5	25.7	4.70	7.75	9.50	1.25	2.02	2.61
BSF–3–4	214	290	3.8	31.3	6.16	9.26	12.26	1.73	2.90	3.17
BSF–3–5	210	276	2.7	30.5	4.88	8.64	11.57	1.44	2.25	3.16
BSF–3–6	204	262	1.6	30.3	4.66	7.90	11.45	1.24	1.97	3.10
BSF–3–7	226	312	2.9	38.2	4.97	9.93	11.24	1.47	2.37	4.33
BSF–3–8	218	282	1.8	35.0	4.75	9.04	11.05	1.26	1.97	3.67
BSF–3–9	212	262	1.2	34.1	4.60	8.29	10.30	1.22	1.86	3.61
D0.5	205	265	11.9	25.0	26.6	50.0	50.40	5.50	9.65	8.20
D1.0	205	265	12.3	25.0	2.02	6.52	8.05	0.45	1.11	1.13
B–1	205	265	13.1	29.0	2.05	5.22	8.11	1.17	2.06	2.77
B–2	205	265	15.1	28.6	2.11	5.68	8.12	1.43	2.32	2.96
B–3	205	265	15.8	27.5	2.17	6.10	8.23	1.58	1.64	3.10
S–1	196	260	5.7	28.4	2.82	6.28	8.25	0.98	1.67	2.66
S–2	184	226	4.2	25.9	3.08	6.62	8.87	1.00	1.63	2.81
S–3	172	206	2.7	24.8	2.78	6.10	8.12	0.93	1.60	2.37
F–1	226	268	6.3	35.0	1.88	5.25	8.10	0.87	1.92	2.14
F–2	238	316	5.4	41.0	2.07	5.47	9.23	0.91	2.00	2.45
F–3	248	318	4.6	45.2	2.10	6.32	9.63	0.98	2.15	2.63

**Table 11:** Orthogonal Experimental Design and Range Analysis of Initial Setting Time.

Test No.	A	B	C	D (Error)	Initial Setting Time/min
1	1	1	1	1	208
2	1	2	2	2	210
3	1	3	3	3	210
4	2	1	2	3	202
5	2	2	3	1	204
6	2	3	1	2	224
7	3	1	3	2	196
8	3	2	1	3	214
9	3	3	2	1	218
Range Analysis					Preferred Combination
K_1_	628	606	646	630	A_2_B_3_C_1_
K_2_	630	628	630	630	
K_3_	628	652	610	626	
k_1_	209.3	202	215.3	210	
k_2_	210	209.3	210	210	
k_3_	209.3	217.3	203.3	208.67	
R	0.67	15.3	12	1.3	
B > C > A					

**Table 12:** Orthogonal Experimental Design and Range Analysis for Final Setting Time.

Test No.	A	B	C	D (Error)	Final Setting Time/min
1	1	1	1	1	264
2	1	2	2	2	276
3	1	3	3	3	266
4	2	1	2	3	248
5	2	2	3	1	264
6	2	3	1	2	314
7	3	1	3	2	218
8	3	2	1	3	290
9	3	3	2	1	282
Range Analysis					Preferred Combination
K_1_	806	730	868	810	A_2_B_3_C_1_
K_2_	826	830	806	808	
K_3_	790	862	748	804	
k_1_	268.7	243.3	289.3	270	
k_2_	275.3	276.7	268.7	269.3	
k_3_	263.3	287.3	249.3	268	
R	12	44	40	2	
B > C > A					

**Table 13:** Range Analysis for Flexural Strength.

Target	NO.	A	B/%	C/%	D/%
3 d Flexural Strength (MPa)	K1	2.61	3.42	3.9	3.37
	K2	3.33	3.65	3.24	3.23
	K3	4.24	3.11	3.04	3.58
	k1	0.87	1.14	1.3	1.12
	k2	1.11	1.22	1.08	1.08
	k3	1.413	1.04	1.01	1.19
	R	0.54	0.18	0.29	0.12
7 d Flexural Strength (MPa)	K1	4.4	5.43	6.32	3.61
	K2	3.55	4.39	5.23	5.29
	K3	6.89	5.02	3.29	5.94
	k1	1.47	1.81	2.11	1.20
	k2	1.18	1.46	1.74	1.76
	k3	2.30	1.68	1.10	1.98
	R	1.11	0.35	1.01	0.78
28 d Flexural Strength (MPa)	K1	7.31	6.74	8.63	8.38
	K2	8.42	8.32	8.24	8.51
	K3	9.45	10.12	8.31	8.29
	k1	2.44	2.25	2.88	2.79
	k2	2.81	2.77	2.75	2.84
	k3	3.15	3.37	2.77	2.76
	R	0.71	1.13	0.13	0.07

**Table 14:** Range Analysis for Compressive Strength.

Target	NO.	A	B/%	C/%	D/%
3 d Compressive Strength (MPa)	K1	11.01	12.93	14.39	12.84
	K2	12.7	14.12	12.85	12.8
	K3	15.61	12.27	12.08	13.68
	k1	3.67	4.31	4.80	4.28
	k2	4.233	4.71	4.28	4.27
	k3	5.20	4.09	4.027	4.56
	R	1.53	0.62	0.77	0.29
7 d Compressive Strength (MPa)	K1	20.45	21.94	23.89	23.17
	K2	22.25	23.34	23.18	22.33
	K3	26.05	23.47	21.68	23.25
	k1	6.82	7.31	7.96	7.72
	k2	7.42	7.78	7.73	7.44
	k3	8.68	7.823	7.23	7.75
	R	1.87	0.51	0.74	0.31
28 d Compressive Strength (MPa)	K1	27.42	27.08	30.95	30.48
	K2	30.08	33.24	30.18	29.74
	K3	32.81	29.99	29.18	30.09
	k1	9.14	9.03	10.32	10.16
	k2	10.03	11.08	10.06	9.91
	k3	10.94	10.00	9.73	10.03
	R	1.80	2.05	0.59	0.25

**Table 15:** Test–Related Content.

Influencing Factor	Range or Parameter
Cement Material Content	10%, 15%, 20%, 25%, 30%
Cement Material Type	Basalt Fiber Solidified Cement Material
Curing Age	7 d, 28 d, 90 d

**Table 16:** Total Cost of Bored Piles Combined with Waterproof Curtain.

No.	Material Type	Unit	Unit Price (Yuan)	Quantity	Cost (Yuan)
1	Concrete C30	m^3^	360	2545.36	916,330
2	HPB300	ton	3570	39.962	142,665
3	HRB335	ton	3870	199.81	773,268
4	Steel Q235	ton	3860	521.42	2,012,682
5	Earthwork	m^3^	20	47,850	957,000
Total					4,801,945

Adjusted total project cost = 4,801,945 × 1.00 = 4,801,945 Yuan = 480.195 (10k Yuan).

**Table 17:** Total Cost of CSM Wall with Embedded H–beams.

No.	Material Type	Unit	Unit Price (Yuan)	Quantity	Cost (Yuan)
1	Concrete C30	m^3^	360	130.733	47,064
2	HPB300	ton	3570	2.053	7327
3	HRB335	ton	3870	10.263	39,716
4	Steel Q345	ton	3860	97.665	376,988
5	CSM Method Steel Section	ton	4230	410.194	1,735,121
6	Cement–Soil	m^3^	200	3608.273	721,655
7	Earthwork	m^3^	20	47,849.984	957,000
Total					3,884,871

Adjusted total project cost = 3,884,871 × 1.00 = 3,884,871 (Yuan) = 388.49 (10k Yuan).

## Data Availability

The original contributions presented in this study are included in the article. Further inquiries can be directed to the corresponding author.
